# The Surgical Anatomy of the Perineum

**Published:** 1839-07

**Authors:** 


ART. IX.—*The Surgical Anatomy of the Perineum.* By THOMAS MORTON, formerly one of the House-Surgeons of the University College Hospital. *Illustrated with Lithographic Plates and Wood Engravings.—London,* 1838. 8vo, pp. 80.

IT may seem ill-natured on our part to find fault with a treatise on which a great deal of care has evidently been bestowed; but as the present is another endeavour to make anatomical knowledge easy, we consider it obnoxious to the objections formerly made by us in noticing works of a similar class: we cannot but have our apprehensions concerning any means resorted to for facilitating the acquirement of anatomy at the expense of actual dissection. We are well aware that Mr. Morton only intends his work to be employed as an aid to the student in pursuing his dissections; but our experience has taught us to place but little reliance on the practical disposition of the idly-disposed, where the temptation of *getting-up* their anatomy from plates is thrown in their way.

Mr. Morton is true to the promise of his title-page, in giving the surgical anatomy of the perineum, and indeed, in great measure, of the pelvis likewise. He seems to have freely consulted the most recent French authorities; and we could wish that he had extended his enquiries to the German anatomists: Müller, in particular, has been occupying himself in minute dissections, both human and comparative, of the male generative organs; and his results, as connected with the muscular structure of the urethra, the arterial distribution in the corpora cavernosa, and the nervous plexus in these parts, present much that is interesting and important. The lithographic plates with which Mr. Morton's work is illustrated are nicely coloured, and, though rather stiff, are on the whole accurate. If then, the student will agree to receive it on our terms, viz. that he shall not abate one stroke of his knife because he has such assistance presented to him, we most cordially recommend Mr. Morton's treatise, as a satisfactory guide in the dissection of the perineum and pelvis.

